# Early-life dentine manganese concentrations and intrinsic functional brain connectivity in adolescents: A pilot study

**DOI:** 10.1371/journal.pone.0220790

**Published:** 2019-08-14

**Authors:** Erik de Water, Demetrios M. Papazaharias, Claudia Ambrosi, Lorella Mascaro, Emilia Iannilli, Roberto Gasparotti, Roberto G. Lucchini, Christine Austin, Manish Arora, Cheuk Y. Tang, Donald R. Smith, Robert O. Wright, Megan K. Horton

**Affiliations:** 1 Icahn School of Medicine at Mount Sinai, New York, NY, United States of America; 2 ASST Spedali Civili Hospital, Brescia, Italy; 3 Technical University Dresden, Germany; 4 University of Brescia, Brescia, Italy; 5 University of California Santa Cruz, Santa Cruz, CA, United States of America; Wayne State University, UNITED STATES

## Abstract

Maturational processes in the developing brain are disrupted by exposure to environmental toxicants, setting the stage for deviant developmental trajectories. Manganese (Mn) is an essential nutrient that is neurotoxic at high levels of exposure, particularly affecting the basal ganglia and prefrontal cortex. Both the intensity and timing of exposure matter; deciduous teeth can be used to retrospectively and objectively determine early-life windows of vulnerability. The aim of this pilot study was to examine associations between prenatal, early postnatal and childhood dentine Mn concentrations and intrinsic functional connectivity (iFC) of adolescents’ brains. 14 adolescents (12–18 years; 6 girls) from northern Italian regions with either current, historic or no Mn contamination, completed a 10-minute resting state functional Magnetic Resonance Imaging (MRI) scan in an 1.5T MRI scanner. We estimated prenatal, early postnatal and childhood Mn concentrations in deciduous teeth using laser ablation-inductively coupled plasma-mass spectrometry. We performed seed-based correlation analyses, focusing on six subcortical seeds (left and right caudate, putamen, pallidum) and one cortical seed (bilateral middle frontal gyrus) from Harvard-Oxford atlases. We examined linear and quadratic correlations between log-transformed Mn concentrations and seed-based iFC (Bonferroni-corrected *p*<0.0023), controlling for either socio-economic status, sex or age. Dentine Mn concentrations (Mn:Calcium ratio) were highest during the prenatal period (median = 0.48) and significantly declined during the early postnatal (median = 0.14) and childhood periods (median = 0.006). Postnatal Mn concentrations were associated with: 1) increased iFC between the middle frontal gyrus and medial prefrontal cortex; 2) decreased iFC between the right putamen and pre- and postcentral gyrus. Together, these findings suggest that early postnatal Mn concentrations are associated with increased iFC within cognitive control brain areas, but decreased iFC between motor areas in adolescents. Future studies should replicate these findings in larger samples, and link brain connectivity measures to cognitive and motor outcomes.

## Introduction

Manganese (Mn) is a prevalent trace metal that is essential for adequate bone growth and enzyme function [1]. Mn is widely used in ferroalloy industries resulting in occupational exposure in adults, while the general population is exposed to Mn through inhalation, dietary intake (e.g., leafy vegetables, nuts, bread and tea) and drinking of contaminated water [2, 3]. Mn is an essential nutrient at low levels of exposure, but it is neurotoxic at levels outside of the homeostatic range, disrupting dopamine and astrocyte function and increasing oxidative stress [4]. The developing brain is particularly vulnerable to Mn exposure. Mn can readily pass placental and blood-brain barriers [5–7]. Given that optimal brain development requires complex maturational processes (e.g., neurogenesis, migration, specialization, myelination, apoptosis) to occur in the right order and at the right time, disruption of these processes by prenatal and early postnatal Mn exposure can override normal brain development towards a maladaptive phenotype, with lasting consequences [8, 9].

Relatively high prenatal, early postnatal and childhood Mn concentrations have been associated with impaired motor function [10, 11], impaired visual spatial performance [12] and decreased IQ [13, 14] and learning and memory [15] in children and adolescents. Only a handful of studies have examined the neural mechanisms of early-life Mn exposure [2, 16–19]. Identifying these neural mechanisms would improve our mechanistic insights into how early-life Mn exposure is linked to poor cognitive and motor outcomes in adolescence, which could inform prevention and intervention efforts [20]. Prior studies have shown that early-life Mn concentrations are particularly associated with the function and structure of brain areas implicated in motor and cognitive control, including the basal ganglia and prefrontal cortex. Aschner and colleagues [16] showed that Mn accumulates in several basal ganglia structures in infants, including the pallidum and putamen (but see ref [2] for contrasting findings in children). Children who consumed drinking water containing higher Mn concentrations showed enlarged putamen volumes compared to children with drinking water with less Mn, and larger putamen volumes were associated with worse fine motor performance [19]. We demonstrated that children with higher prenatal maternal blood Mn concentrations showed reduced intrinsic functional connectivity between the medial and lateral prefrontal cortex and the pallidum [17]. In addition, Iannilli et al. [18] reported reduced prefrontal cortex activity during an odor stimulation task in adolescents from an Mn contaminated area.

Previous neuroimaging studies have used spot measures of Mn exposure, such as concentrations in maternal blood or drinking water [2, 17]. In addition to not measuring fetal and childhood exposure directly [21], these measures also do not allow one to investigate vulnerability windows during which exposure is particularly harmful. This may lead to missed associations, as it has been shown that associations between early-life Mn concentrations and neurodevelopment in children and adolescents do not only depend on the intensity but also on the timing of exposure [12, 15]. Recently, we have developed and validated deciduous teeth biomarkers of Mn exposure to overcome these limitations [22–24]. During the second trimester of pregnancy, dentine begins to be deposited in a rhythmic manner (creating incremental lines akin to tree growth rings), which continues until the tooth is shed (between ~6–12 years) [25]. At birth, a distinct histological landmark—the neonatal line—is formed, which can be used to distinguish prenatally from postnatally formed parts of the tooth [25]. Deciduous teeth accumulate metals, including Mn [22], and can therefore be used to reconstruct both the intensity and timing of Mn exposure throughout pregnancy and childhood. Moreover, teeth biomarkers reflect direct fetal exposure, unlike other biomarkers such as maternal blood during pregnancy [21].

The aim of this pilot study was to examine how prenatal, early postnatal and childhood dentine Mn concentrations in deciduous teeth are associated with intrinsic functional connectivity of the brain in adolescents. Intrinsic functional connectivity reflects correlations between spontaneous activity of different brain areas at rest [26], and can be used to examine connectivity between networks across the brain, instead of focusing on isolated brain areas. Based on prior studies showing associations between early-life Mn concentrations and impaired cognitive and motor control in adolescents [10–15], we hypothesized that adolescents with higher Mn concentrations would show functional connectivity patterns that are characteristic of individuals with poor cognitive and motor control [27–29]. Specifically, we expected adolescents with higher early-life Mn concentrations to show reduced intrinsic functional connectivity between the basal ganglia and prefrontal cortex [17], reduced connectivity between the prefrontal and parietal cortex, and reduced connectivity between the basal ganglia and motor cortex.

## Materials and methods

### Participants

Participants were part of an ongoing cohort study called PHIME (Public Health Impact of Manganese Exposure) in the Province of Brescia in Northern Italy [10, 11, 15, 30]. Upon enrollment, PHIME participants reported living in three geographically distinct, but demographically similar, areas with varying levels of Mn contamination [30]: 1) Bagnolo Mella, with an active ferromanganese plant; 2) Valcamonica, with historic ferromanganese plant activity (the last plant was closed in 2001); 3) Garda Lake, with no history of ferromanganese plant activity. Enrollment and inclusion and exclusion criteria for the PHIME study are described in detail elsewhere [11]. Briefly, adolescents were enrolled through the public school system according to a community-based participatory approach that involved the local communities of the three sites. The inclusion criteria included: 1) to be born in the study area from resident families living in the study area since the 1970s; 2) to live in the study area since birth; 3) to be aged 11–14 years at enrollment. The exclusion criteria were: 1) having a neurological, hepatic, metabolic, endocrine or psychiatric disorder; 2) use of medications with known neuropsychological side-effects; 3) having clinically diagnosed motor deficits or cognitive impairment; 4) having visual deficits that are not adequately corrected. Upon enrollment into PHIME, all participants completed baseline questionnaires, including information on family socioeconomic status [31]. Participants also completed neuropsychological tests, including measures of IQ (Wechsler Intelligence Scale for Children, 3^rd^ edition; WISC-III) [32], memory and motor function. A subset of PHIME participants (*n* = 211) provided deciduous teeth, which were used to determine Mn exposure throughout pregnancy [10, 15]. For the present pilot neuroimaging study [18], 14 adolescents (6 girls, 12–18 years, *M* age = 14.63 years, *SD* = 2.12 years) who had provided deciduous teeth were invited from the PHIME cohort to participate in a multi-modal Magnetic Resonance Imaging (MRI) study. Participants of this pilot study were living in Bagnolo Mella (*n* = 7), Valcamonica (*n* = 3) or Garda Lake (*n* = 4) regions. Written informed consent was obtained from parents, while participants provided written assent. Study procedures were approved by the ethical committees of the University of Brescia, and the Icahn School of Medicine at Mount Sinai.

### Dentine manganese concentrations

Participants provided their naturally shed deciduous teeth. Incisors, canines and molars that were free of defects such as caries and extensive tooth wear were analyzed. Analysis methods have been validated and described in detail previously [22]. Briefly, teeth were washed in an ultrasonic bath of ultrapure Milli-Q water (18.2 Mohm-cm^2^) and dried before and after sectioning. Teeth were sectioned on a vertical plane using a diamond encrusted blade. The neonatal line, a histological landmark distinguishing pre- and postnatally formed regions of dentine, was identified under light microscopy. With the neonatal line as a reference point, the concentrations and spatial distribution of Mn in different developmental windows were determined using laser ablation inductively coupled plasma mass spectroscopy (LA-ICP-MS; Agilent QQQ 8800 coupled with ESI 193nm laser ablation unit). Prenatally formed primary dentine adjacent to the enamel-dentine junction was sampled to determine *prenatal Mn concentrations*; postnatally formed primary dentine after the neonatal line was sampled to determine *early postnatal Mn concentrations*, which reflect exposure up to the first year of life. Measurements in secondary dentine, formation of which starts after the completion of the tooth root and proceeds at a slower rate as long as the tooth remains vital, were used to estimate *childhood cumulative Mn concentrations* from root completion to the time the tooth was shed. Dentine Mn concentrations were normalized to dentine calcium levels (^55^Mn:^43^Ca ratio) to account for variations in mineral density within and between teeth [22]. Multiple measurements were taken in prenatal and postnatal dentine; the area under the curve (AUC) of Mn concentrations across all sampling points was computed to estimate concentrations during each developmental period. The limit of detection was 0.02 μg/g. Values below the detection limit (*n* = 1 for early postnatal Mn) were assigned half of the lowest value among the samples above the detection limit.

### Neuroimaging data acquisition

A 10-minute resting state functional MRI (fMRI) scan was acquired using a 1.5T Magnetom Aera MRI scanner (Siemens, Erlangen, Germany) equipped with a 20-channel phased array head-neck coil, at the Civil Hospital of Brescia, Italy. We used a 2D echo planar imaging (EPI) sequence (TR = 2500 ms, TE = 50 ms, 240 volumes, 29 slices, 3.5 mm thickness, 1.1 mm inter-slice gap, matrix size = 64 x 64) sensitive to blood-oxygen-level dependent (BOLD) contrast. During this acquisition, lights were turned off and subjects were instructed to keep their eyes closed, not to think of anything in particular, and to not fall asleep. For registration purposes, we acquired a 3D magnetization-prepared rapid gradient echo (MPRAGE) T1-weighted scan (TR = 2040 ms, TE = 3.08 ms, Field of View = 256 mm, matrix size = 320 x 320, 144 slices, voxel size = 1 mm^3^).

## Statistical analyses

### Descriptive statistics

Descriptive statistical analyses were performed using SPSS version 22. Dentine Mn and covariate data were examined for normality. Differences between the pilot participants (*n* = 14) and the parent study (*n* = 717) were examined using Chi-square tests (for categorical variables) and independent samples t-tests (for continuous variables). Dentine Mn was natural log-transformed to reduce skewness and approximate a normal distribution. Changes in dentine Mn concentrations from the prenatal period to childhood were examined using a repeated-measures ANOVA. Correlations between dentine Mn at three time-points were examined using Pearson correlation coefficients. Differences in Mn concentrations and IQ between the three geographic locations in which participants reported living were examined using One Way ANOVA.

### Preprocessing of resting state fMRI data

Preprocessing was carried out using FEAT [33] Version 6.00, part of FSL (FMRIB's Software Library, www.fmrib.ox.ac.uk/fsl). The first two volumes were discarded to allow for T1-equilibration effects. We performed motion correction using MCFLIRT [34], slice-timing correction using Fourier-space time-series phase-shifting, non-brain removal using BET [35], spatial smoothing using a Gaussian kernel of 6 mm full-width at half maxium (FWHM), and highpass temporal filtering (Gaussian-weighted least-squares straight line fitting, with sigma = 100.0 s). Registration of functional images to participants’ high resolution structural images was carried out using FLIRT [34, 36], and we used FLIRT and FNIRT nonlinear registration [37, 38] to register participants’ high resolution structural images to standard space (MNI-152). The transformations were then applied to the functional images to register them to standard space.

### Seed-based correlation analyses

**Fig 1:**
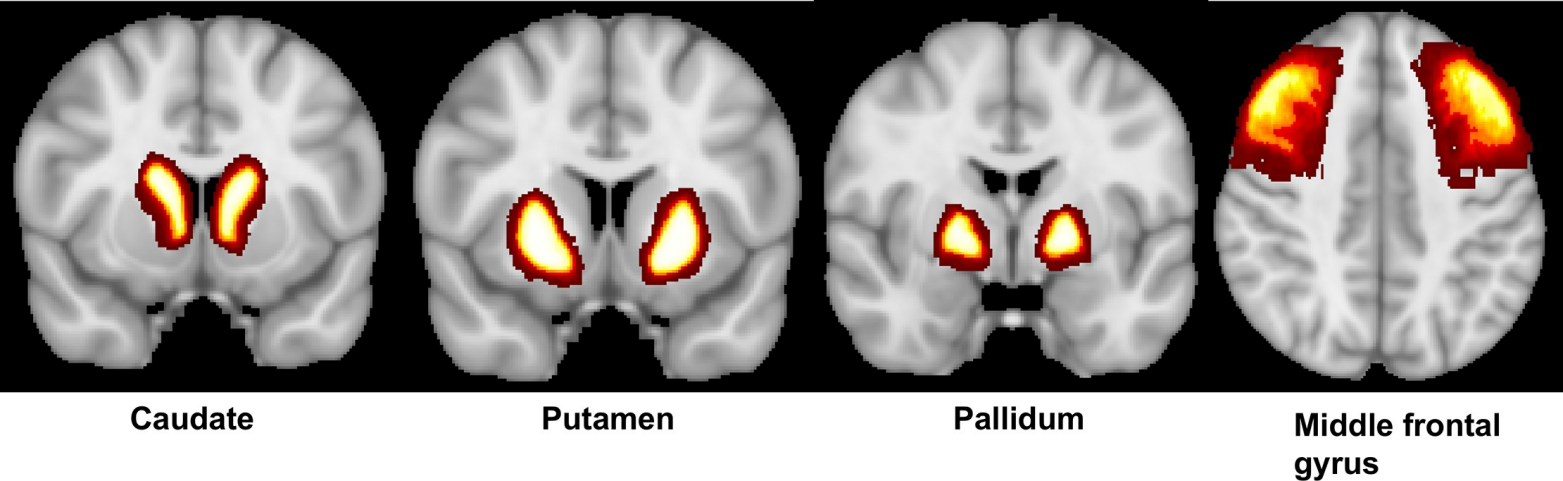
Seeds used in seed-based correlation analyses. The following 7 seeds were selected from the Harvard-Oxford subcortical (seeds 1–6) and cortical (seed 7) atlases: 1) Left Caudate; 2) Right Caudate; 3) Left Putamen; 4) Right Putamen; 5) Left Pallidum; 6) Right Pallidum; 7) Bilateral Middle Frontal Gyrus.

We used FSL’s Featquery to extract the mean timeseries of seven seed regions in participant’s native space focusing on brain regions shown to be affected by prenatal and early-life Mn exposure in prior research [2, 16–18]. Specifically, we selected six seeds from the probabilistic Harvard-Oxford Subcortical Structural Atlas [39]: left putamen (19805 mm^3^), right putamen (20057 mm^3^), left caudate (16087 mm^3^), right caudate (17126 mm^3^), left pallidum (9565 mm^3^) and right pallidum (9932 mm^3^). We further selected the bilateral middle frontal gyrus seed (143253 mm^3^) from the probabilistic Harvard-Oxford Cortical Structural Atlas. All seeds are shown in Fig 1. Using FEAT, the extracted timeseries of each seed was included as a predictor in a lower-level multiple regression analysis for each participant and seed separately, which produced Z-value correlation maps of all voxels that positively and/ or negatively correlated with the seed timeseries. This analysis was carried out using FILM with local autocorrelation correction [40]. We included the following nuisance regressors in these lower-level multiple regression analyses for each participant: 24 motion parameters [41] and the mean timeseries of white matter (WM) and cerebrospinal fluid (CSF). We used FSL’s FAST to segment the 3D MPRAGE image into WM and CSF, and used Featquery to extract the mean timeseries of the thresholded (probability = 0.8) WM and CSF images. Additionally, we used the fsl_motion_outliers tool to compute relative framewise displacement (FD), and regressed out volumes that were corrupted by excessive motion (FD > 0.2 mm) [42]. These noise and motion confounders were regressed out in the lower-level multiple regression analyses for each participant, before any group-level analyses were carried out. Group-level analyses were carried out using a mixed-effects model implemented in FSL FLAME (stage 1). The general linear model to examine exposure-related changes in the brain included the mean-centered linear and quadratic effects of Mn exposure as predictors. Statistical images were thresholded using clusters determined by Z > 2.3 and cluster-corrected (using Gaussian Random Field theory) threshold of *p* < 0.05 [17, 43–45]. Given that prenatal, early postnatal and childhood Mn concentrations were not significantly correlated (*r*’s .12-.22; all *p*’s > .46), we performed separate analyses for each of the three timepoints. In order to control for the number of comparisons (*n* = 21; 7 seed regions * 3 Mn timepoints), we applied a Bonferroni-correction (*p* = .05/21 = 0.00238) and only report findings surviving this Bonferroni-correction (cluster-corrected *p* < .00238).

### Covariates

Given that age, sex and socio-economic status (SES) may impact intrinsic functional connectivity [46–48] and associations between Mn exposure and neurodevelopment [10, 49], we included these variables as covariates (one at a time) in the group-level analyses. SES was determined as either low, medium or high, based on parental education and occupation [31].

## Results

### Demographic characteristics, Mn concentrations and head motion

Most participants lived in areas with either current (50%) or historic (21.4%) Mn contamination due to ferroalloy plant activity. Most participants came from a medium SES background (50%), but low (21.4%) and high SES (28.6%) backgrounds were represented as well. Participants of the neuroimaging pilot study (*n* = 14) did not differ significantly (all *p*’s > 0.31) from the full PHIME cohort (*n* = 717) in terms of child sex, IQ, SES, geographic location or dentine Mn concentrations (see Table 1).

**Table 1:** Demographic and exposure characteristics of the participants of the pilot resting state fMRI study described here (n = 14) and of the PHIME cohort study participants who were not included in the pilot resting state fMRI study (n = 717).

Characteristic	Pilot neuroimaging study (n = 14) Median (IQR) or %	Full PHIME cohort (n = 717) Median (IQR) or %	*p* ^*a*^
**SES** (%) Low/Medium/High	21.4/50.0/28.6	23.8/53.0/23.1	0.97
**Child sex** (% Female)	42.9	48.1	0.92
**IQ**	109 (19.3)	108 (17)	0.32
**Geographic location** (%) BM/VC/GL	50.0/21.4/28.6	29.7/34.4/35.8	0.42
**Dentine Mn concentrations** (Mn:Ca)^b^ Prenatal/Early postnatal/Childhood	0.48(0.25)/0.14(0.08)/0.0006(0.0005)	0.43(0.20)/0.13(0.07)/0.0007(0.0004)	0.63/0.87/0.56

*Note*. Mn = manganese; Ca = calcium; SES = socio-economic status; IQ was measured with the Wechsler Intelligence Scale for Children, 3^rd^ edition (WISC-III); BM = Bagnolo Mella; VC = Valcamonica; GL = Garda Lake

^a^ Differences in the distribution of categorical variables were tested using Chi-Square tests, while differences in continuous variables were compared using independent samples t-tests.

^b^ Dentine Mn concentrations were available for 197 out of the 717 participants of the full PHIME cohort.

Dentine Mn concentrations significantly declined from the prenatal period to childhood (*F*(2,24) = 96.82, *p* <0.001, ηp^2^ = 0.89). Specifically, prenatal concentrations were higher than early postnatal (*p* <0.001) and childhood concentrations (*p* <0.001), and early postnatal concentrations were higher than childhood concentrations (*p* <0.001). Mn concentrations (all *p*’s > 0.12) and IQ (*p* > 0.82) did not differ significantly between participants who reported living in areas with current, historic or no exposure.

Head motion was low overall: the mean relative framewise displacement (FD) of all scans included in the analyses was 0.06 mm. The mean relative FD was not significantly correlated with Mn concentrations (*p*’s >.22).

### Seed-based correlation analyses

**Fig 2:**
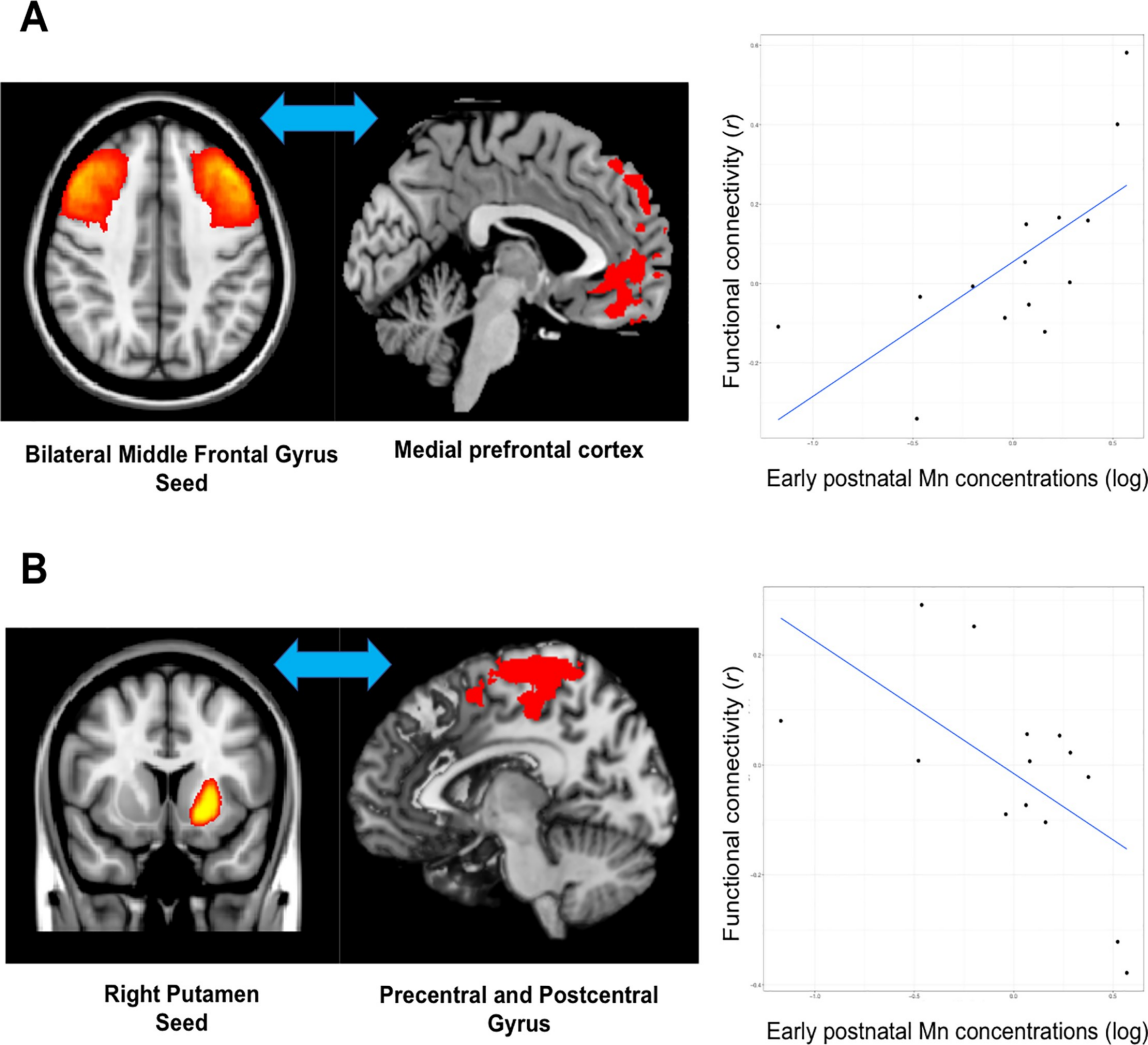
Results of seed-based correlation analyses. A) positive correlation (cluster-corrected *p*<0.00238) between natural log-transformed early postnatal Mn concentrations and functional connectivity between the bilateral middle frontal gyrus and the left medial prefrontal cortex; B) negative correlation (cluster-corrected *p*<0.00238) between natural log-transformed early postnatal Mn concentrations and functional connectivity between the right putamen and the left precentral and postcentral gyrus. *Note*. Mn = manganese. The Mn:Ca ratio was used to quantify concentrations to account for mineral variation between and within teeth.

We found a positive correlation between early postnatal dentine Mn concentration and functional connectivity between the bilateral middle frontal gyrus seed and a large cluster in the left medial prefrontal cortex (10140 mm^3^, Z = 4.05, *p* <0.0001, MNI coordinates of peak voxel: X = -5, Y = 50, Z = 2), extending into the ventral anterior cingulate cortex, frontal pole and superior frontal gyrus (see Fig 2A). Adolescents with higher early postnatal Mn concentrations thus showed increased functional connectivity between the bilateral middle frontal gyrus and left medial prefrontal cortex.

We found a negative correlation between early postnatal dentine Mn concentration and functional connectivity between the right putamen seed and a large cluster in the left precentral and postcentral gyrus (31986 mm^3^, Z = 3.80, *p* <0.0001, MNI coordinates of peak voxel: X = -15, Y = -26, Z = 76), extending into the supplementary motor area, precuneus and superior parietal lobule (see Fig 2B). Adolescents with higher early postnatal Mn concentrations thus showed reduced functional connectivity between the right putamen and left pre- and postcentral gyrus.

These findings remained significant after controlling separately for either SES, child sex or age. We did not observe associations between prenatal dentine Mn or childhood dentine Mn and intrinsic functional connectivity among these subjects.

## Discussion

The aim of this pilot study was to examine how prenatal, early postnatal and childhood dentine Mn concentrations are associated with intrinsic functional connectivity of the brain in adolescents. Our main findings were that adolescents with higher early postnatal Mn concentrations showed: 1) *increased* intrinsic functional connectivity between the middle frontal gyrus and medial prefrontal cortex; and 2) *decreased* connectivity between the right putamen and the pre- and postcentral gyrus.

These findings are consistent with our prior study in which we showed that higher prenatal Mn concentrations measured in maternal blood were associated with reduced intrinsic functional connectivity of the basal ganglia and prefrontal cortex in children [17]. Other studies focusing on brain volume also showed that the basal ganglia is affected by early-life Mn exposure [19], and the volume of the prefrontal cortex is reduced in those with higher early-life exposure to other metals (i.e., lead) as well [50]. The prefrontal cortex is implicated in cognitive control [51]; increased intrinsic functional connectivity (i.e., reduced anticorrelations) between the lateral and medial prefrontal cortex has been linked to poor working memory [28]. The putamen is involved in motor control [52]; reduced intrinsic functional connectivity of the putamen and precentral gyrus was observed in children with impaired motor function [29]. Taken together, our findings thus suggest that higher early postnatal Mn concentrations are associated with intrinsic functional connectivity patterns that are typically observed in individuals with poor cognitive and motor control. This is in line with previous research demonstrating that higher early postnatal dentine Mn concentrations are associated with poor cognitive [12] and motor control [10] in adolescents.

Mn can readily pass placental and blood-brain barriers [5–7] and may exert its neurotoxic effects by disrupting dopamine function, increasing oxidative stress and disrupting the function of astrocytes, which are critical for the development of structural connections underlying the brain’s functional connectivity [4].

In the present study, only early postnatal Mn concentrations were associated with intrinsic functional connectivity of adolescents’ brains, after controlling for multiple comparisons. This is consistent with two recent studies showing that the early postnatal period may be a critical window of vulnerability to the effects of Mn exposure on neurodevelopment [12, 15, 53]. The early postnatal period may be a window of heightened vulnerability to Mn exposure, because contrary to the prenatal period, Mn homeostasis is no longer tightly regulated by the placenta, while the infants’ blood-brain barrier and ability to regulate Mn is less developed than in later childhood [12].

Prenatal and early postnatal dentine Mn concentrations in the current study were comparable to those reported in the CHAMACOS study in California [21, 49], which used a similar method to determine dentine Mn concentrations. In the CHAMACOS study, the main Mn exposure source was the use of Mn-containing fungicides within the agricultural community in which children were raised. In the province in Northern Italy in which participants of our pilot study are residing, the main Mn exposure sources have been shown to be ingestion of soil [54], ingestion of dust [30], and inhalation of air [55] contaminated by emissions from ferromanganese alloy plants.

A major strength of this pilot study is the use of deciduous teeth to measure dentine Mn concentrations. Dentine Mn concentrations measure direct fetal exposure, unlike other commonly used biomarkers such as maternal blood [21]. Further, using teeth as a biomarker allowed us to objectively and retrospectively measure Mn concentrations during pregnancy, the early postnatal period and childhood, which enabled us to identify the early postnatal period as a critical developmental window for effects of Mn exposure on intrinsic functional connectivity of the brain. To our knowledge, this study was the first to combine a neuroimaging method (i.e., a measure of intrinsic functional connectivity) with a measure of early-life dentine Mn concentrations in deciduous teeth. This may provide detailed insights into the neural mechanisms that may underpin associations between early-life Mn exposure and cognitive and motor control reported in prior studies [10, 12, 15]. Prevention programs should be developed to reduce early-life Mn exposure, while neurocognitive interventions (e.g., cognitive training, neurofeedback; [56, 57] targeting brain areas affected by Mn exposure may reduce its harmful effects.

Several limitations of the present study deserve to be mentioned as well. While the associations we observed were robust, in that they survived controlling for multiple comparisons and confounders (i.e., age, sex, SES), the sample size of this pilot study was modest. We may have been unable to detect non-linear associations between Mn concentrations and brain connectivity, due to this modest sample size. Future studies need to replicate our findings with a larger sample size, and include measures of cognitive and motor control to test whether intrinsic functional connectivity patterns associated with Mn concentrations are further associated with cognitive and motor control. Future investigations may also include measures of brain volume, to explore whether changes in functional connectivity can be explained by changes in brain volume associated with early-life Mn exposure. A larger sample size would further allow one to explore associations stratified by sex, given that associations between Mn exposure and cognitive and motor control have been shown to be sex-dependent [10, 12, 49]. Future research should further examine associations between co-exposure to mixtures of metals (e.g., manganese, lead, zinc, cadmium) and intrinsic functional connectivity of the brain.

To conclude, we found that early postnatal Mn concentrations measured in deciduous teeth were associated with altered intrinsic functional connectivity of brain areas involved in cognitive and motor control in adolescents. Prevention programs should be implemented to reduce early-life Mn exposure, while neurocognitive intervention programs could be developed to reduce the harmful consequences in adolescents who were exposed to high Mn levels during pregnancy, infancy and early-childhood.
